# Supplementary material for: Genetic Diversity of Candida spp. Isolates Colonizing Twins and Their Family Members
Source: Pathogens. 2022 Dec 13;11(12):1532. doi: 10.3390/pathogens11121532 (PMC9783311; doi:10.3390/pathogens11121532)
Supplement: Supplementary file 1 [file pathogens-11-01532-s001.zip › pathogens-1988702-supplementary.pdf]

Table S1. The names and sequences of the primers used for random amplified polymorphic DNA – polymerase chain reaction (RAPD-PCR) analysis for each *Candida* sp.

No.	*Candida* sp.	Primer	Sequence 5′ – 3′
1.	*C. albicans*	1247	AAGAGCCCGT
2.		1290	GTGGATGCGA
3.	*C. dubliniensis*	SOY	AGGTCAGTGA
4.		1247	AAGAGCCCGT
5.	*C. parapsilosis*	RP2	AAGGATCAGA
6.		1247	AAGAGCCCGT
7.	*C. guilliermondii*	1247	AAGAGCCCGT
8.		1290	GTGGATGCGA
9.	*C. krusei*	SOY	AGGTCAGTGA
10.		RP2	AAGGATCAGA

Table S2. A detailed summary of the study group and results obtained in this study.

No.	No. of family	Age	Family member	*Candida* sp.	Biochemical tests: Germ tube	Biochemical tests: ATB ID 32C	Source	Name of strain	Genotype/Unique pattern (Un)
1.	F1	42	Mother	*C. albicans*	POS	7349350215	Oral cavity	F1_M_OC1_CA	A4
1.	F1	42	Mother	*C. albicans*	POS	7349350215	Oral cavity	F1_M_OC2_CA	A4
2.	F1	12	Twin 1	*C. albicans*	POS	7347350215	Oral cavity	F1_T1_OC_CA	A3
2.	F1	12	Twin 1	*C. krusei*	NEG	0300010001	Anus	F1_T1_A_CK	A1
2.	F1	12	Twin 1	*C. krusei*	NEG	0300010001	Oral cavity	F1_T1_OC_CK	A1
3.	F1	12	Twin 2	*C. albicans*	POS	7347350215	Oral cavity	F1_T2_OC_CA	A4
3.	F1	12	Twin 2	*C. albicans*	POS	7347350215	Anus	F1_T2_A_CA	A4
4.	F2	29	Father	*C. albicans*	POS	7347340015	Oral cavity	F2_F_OC_CA	A2
5.	F2	32	Mother	*C. albicans*	POS	7347350215	Oral cavity	F2_M_OC1_CA	B
5.	F2	32	Mother	*C. albicans*	POS	7347340215	Oral cavity	F2_M_OC2_CA	B
5.	F2	32	Mother	*C. albicans*	POS	7347350215	Anus	F2_M_A_CA	B
5.	F2	32	Mother	*C. parapsilosis*	NEG	5507360317	Interdigital space	F2_M_IS_CP	E
6.	F2	6	Twin 2	*C. albicans*	POS	7347350215	Oral cavity	F2_T2_OC_CA	B
6.	F2	6	Twin 2	*C. albicans*	POS	7347350215	Anus	F2_T2_A_CA	B
6.	F2	6	Twin 2	*C. guilliermondii*	NEG	5577352117	Oral cavity	F2_T2_OC_CG	B2
6.	F2	6	Twin 2	*C. guilliermondii*	NEG	5577352117	Anus	F2_T2_A_CG	B2
6.	F2	6	Twin 2	*C. guilliermondii*	NEG	5577352117	Interdigital space	F2_T2_IS_CG	B2
6.	F2	6	Twin 2	*C. parapsilosis*	NEG	5547350317	Anus	F2_T2_A_CP	E
7.	F3	46	Mother	*C. albicans*	POS	7347350015	Oral cavity	F3_M_OC1_CA	A2
7.	F3	46	Mother	*C. albicans*	POS	7347350015	Oral cavity	F3_M_OC2_CA	A2
7.	F3	46	Mother	*C. albicans*	POS	7347340015	Oral cavity	F3_M_OC3_CA	A2
8.	F3	11	Twin 1	*C. albicans*	POS	7347340015	Anus	F3_T1_A1_CA	A2
8.	F3	11	Twin 1	*C. albicans*	POS	7347340015	Anus	F3_T1_A2_CA	A2
9.	F4*	45	Father	*C. albicans*	POS	7346340015	Oral cavity	F4_F_OC1_CA	C4
9.	F4*	45	Father	*C. albicans*	POS	7347340015	Oral cavity	F4_F_OC2_CA	C4
10.	F4*	43	Mother	*C. albicans*	POS	7347340015	Oral cavity	F4_M_OC1_CA	C4
10.	F4*	43	Mother	*C. albicans*	POS	7347340015	Oral cavity	F4_M_OC2_CA	C4
10.	F4*	43	Mother	*C. albicans*	POS	7347340015	Anus	F4_M_A1_CA	C4
10.	F4*	43	Mother	*C. albicans*	POS	7347340015	Anus	F4_M_A2_CA	C4
11.	F4*	14	Twin 1	*C. albicans*	POS	7347340015	Oral cavity	F4_T1_OC1_CA	C4
11.	F4*	14	Twin 1	*C. albicans*	POS	7347340015	Oral cavity	F4_T1_OC2_CA	C4
12.	F4*	14	Twin 2	*C. albicans*	POS	7347340015	Oral cavity	F4_T2_OC_CA	C4
13.	F5*	40	Mother	*C. albicans*	POS	7347350215	Oral cavity	F5_M_OC_CA	C2
14.	F5*	14	Twin 1	*C. albicans*	POS	7347140015	Oral cavity	F5_T1_OC1_CA	C3
14.	F5*	14	Twin 1	*C. albicans*	POS	7347340015	Oral cavity	F5_T1_OC2_CA	C2
14.	F5*	14	Twin 1	*C. albicans*	POS	7347140015	Oral cavity	F5_T1_OC3_CA	C2

15.			Twin 2	*C. albicans*	POS	7347140015	Oral cavity	F5_T2_OC_CA	C2
					POS	7347140015	Anus	F5_T2_A1_CA	C2
					POS	7347140015		F5_T2_A2_CA	C3
16.	F6*	29	Father	*C. albicans*	POS	7367340015	Oral cavity	F6_F_OC1_CA	D2
					POS	7347340015		F6_F_OC2_CA	D2
					POS	7347340015		F6_F_OC3_CA	D2
					POS	7146340015		F6_F_OC4_CA	D2
					POS	7146340015	Anus	F6_F_A_CA	D2
17.		29	Mother	*C. albicans*	POS	7347140015	Oral cavity	F6_M_OC1_CA	D2
					POS	7347140015		F6_M_OC2_CA	D2
					POS	7346140015		F6_M_OC3_CA	D2
					POS	7347340015		F6_M_OC4_CA	D2
18.		2	Twin 1	*C. albicans*	POS	7347140015	Oral cavity	F6_T1_OC_CA	D2
					POS	7347140015	Anus	F6_T1_A1_CA	C1
					POS	7347140015		F6_T1_A2_CA	C1
19.			Twin 2	*C. albicans*	POS	7347140015	Oral cavity	F6_T2_OC1_CA	C1
					POS	7347140015		F6_T2_OC2_CA	D2
20.	F7	36	Mother	*C. albicans*	POS	7347140015	Oral cavity	F7_M_OC_CA	D1
					POS	7347340015	Navel	F7_M_N_CA	D1
21.		10	Siblings	*C. albicans*	POS	7347340015	Oral cavity	F7_SIB_OC_CA	D1
22.		12	Twin 1	*C. albicans*	POS	7347340015	Oral cavity	F7_T1_OC_CA	D1
23.			Twin 2	*C. albicans*	POS	7347340015	Oral cavity	F7_T2_OC_CA	D1
24.	F8*	11	Siblings	*C. albicans*	POS	7346340015	Oral cavity	F8_B_OC_CA	Un3
				C. parapsilosis	NEG	5547350317	Interdigital space	F8_SIB_ IS_CP	H2
25.		9	Twin 1	*C. albicans*	POS	7347340015	Oral cavity	F8_T1_OC_CA	I
26.			Twin 2	*C. albicans*	POS	7347340015	Oral cavity	F8_T2_OC1_CA	I
					POS	7347340015		F8_T2_OC2_CA	I
				C. parapsilosis	NEG	5547350717	Oral cavity	F8_T2_OC1_CP	H1
					NEG	5547350317		F8_T2_OC2_CP	H1
					NEG	5542350317		F8_T2_OC3_CP	H1
					NEG	5542350317		F8_T2_OC4_CP	H1
27.	F9	34	Mother	*C. albicans*	POS	7347340015	Oral cavity	F9_M_OC_CA	J5
					POS	7347340015	Anus	F9_M_A_CA	J3
				C. parapsilosis	NEG	5547350317	Oral cavity	F9_M_OC_CP	A1
28.		1	Twin 1	*C. albicans*	POS	7147340015	Oral cavity	F9_T1_OC_CA	J4
					POS	7147340015	Interdigital space	F9_T1_ IS_CA	Un2
					POS	7147340015	Anus	F9_T1_A_CA	J5
				C. krusei	NEG	0300010001	Anus	F9_T1_A_CK	C2
29.			Twin 2	*C. albicans*	POS	7347340015	Oral cavity	F9_T2_OC_CA	J4
					POS	7347340015	Anus	F9_T2_A_CA	J4

				C. parapsilosis	NEG	5547350317	Oral cavity	F9_T2_OC_CP	A2
					NEG	5547350317	Anus	F9_T2_A_CP	A2
				C. krusei	NEG	0300010001	Oral cavity	F9_T2_OC_CK	C2
					NEG	0300010001	Anus	F9_T2_A_CK	C1
					NEG	0300010001	Interdigital space	F9_T2_ IS_CK	C3
30.	F10*	41	Mother	*C. albicans*	POS	7347340015	Oral cavity	F10_M_OC_CA	M3
					POS	7147340015	Anus	F10_M_A_CA	M3
					POS	7347340015	Interdigital space	F10_M_ IS_CA	M4
31.		13	Twin 1	*C. albicans*	POS	7347340015	Oral cavity	F10_T1_OC1_CA	M3
					POS	7347340015		F10_T1_OC2_CA	M3
32.			Twin 2	*C. albicans*	POS	7347340015	Oral cavity	F10_T2_OC1_CA	M3
					POS	7347340015		F10_T2_OC2_CA	M3
					POS	7347340015	Anus	F10_T2_A_CA	M3
33.	F11	21	Twin 1	*C. albicans*	POS	7347350215	Oral cavity	F11_T1_OC_CA	M3
34.		21	Twin 2	*C. albicans*	POS	7347350215	Anus	F11_T2_A_CA	M3
35.	F12*	15	Twin 1	*C. albicans*	POS	7347340015	Oral cavity	F12_T1_OC_CA	M2
				C. parapsilosis	NEG	7147350715	Oral cavity	F12_T1_OC1_CP	B1
					NEG	7147350715		F12_T1_OC2_CP	Un2
					NEG	5545300117	Interdigital space	F12_T1_ IS_CP	B2
36.			Twin 2	*C. albicans*	POS	7347340015	Oral cavity	F12_T2_OC1_CA	M2
					POS	7347340015		F12_T2_OC2_CA	M1
				C. parapsilosis	NEG	7147350715	Interdigital space	F12_T2_ IS_CP	B3
37.	F13*	17	Twin 1	*C. albicans*	POS	7346340015	Oral cavity	F13_T1_OC1_CA	J1
					POS	7346340015		F13_T1_OC2_CA	J1
					POS	7346340015	Anus	F13_T1_A1_CA	J1
					POS	7346340015		F13_T1_A2_CA	J1
38.			Twin 2	*C. albicans*	POS	7346340015	Oral cavity	F13_T2_OC_CA	J1
39.	F14	6	Siblings	*C. albicans*	POS	7146340015	Oral cavity	F14_SIB_OC_CA	Un1
40.		2	Twin 1	*C. albicans*	POS	7146340015	Oral cavity	F14_T1_OC_CA	A1
41.			Twin 2	*C. albicans*	POS	7146340015	Interdigital space	F14_T2_ IS1_CA	A1
					POS	7146340015		F14_T2_ IS2_CA	A1
					POS	7146340015		F14_T2_ IS3_CA	F1
42.	F15	39	Mother	*C. parapsilosis*	NEG	5545350117	Oral cavity	F15_M_OC_CP	C
					NEG	5545350117	Interdigital space	F15_M_ IS1_CP	C
					NEG	5545350117		F15_M_ IS2_CP	D2
43.		9	Twin 1	*C. albicans*	POS	7146340015	Oral cavity	F15_T1_OC_CA	M3
				C. parapsilosis	NEG	7147350715	Interdigital space	F15_T1_ IS1_CP	C
					NEG	7147350715		F15_T1_ IS2_CP	Un3
44.			Twin 2	*C. albicans*	POS	7146340015	Oral cavity	F15_T2_OC1_CA	M3
					POS	7146340015		F15_T2_OC2_CA	M3

				C. parapsilosis	NEG	7147350117	Interdigital space	F15_T2_ IS1_CP	D1
					NEG	5545350117		F15_T2_ IS2_CP	D1
					NEG	5545350117		F15_T2_ IS3_CP	D1
45.	F16*	34	Mother	*C. albicans*	POS	7147340015	Oral cavity	F16_M_OC1_CA	F2
					POS	7146340015		F16_M_OC2_CA	F2
					POS	7146340015	Anus	F16_M_A_CA	F2
46.		39	Father	*C. albicans*	POS	7147340015	Oral cavity	F16_F_OC_CA	J2
					POS	7147340015	Interdigital space	F16_F_ IS_CA	J2
47.		11	Twin 1	*C. albicans*	POS	7147340015	Oral cavity	F16_T1_OC1_CA	J2
					POS	7146340015		F16_T1_OC2_CA	F1
					POS	7147340015		F16_T1_OC3_CA	F2
					POS	7147340015		F16_T1_OC4_CA	F2
48.			Twin 2	*C. albicans*	POS	7147340015	Oral cavity	F16_T2_OC_CA	J2
49.	F17	35	Father	*C. krusei*	NEG	0300010001	Oral cavity	F17_F_OC_CK	B2
50.		13	Twin 1	*C. parapsilosis*	NEG	5547350717	Oral cavity	F17_T1_OC_CP	Un1
				C. krusei	NEG	0300010001	Oral cavity	F17_T1_OC_CK	B1
51.			Twin 2	*C. parapsilosis*	NEG	7547350317	Interdigital space	F17_T2_ IS1_CP	A1
					NEG	7347340015		F17_T2_ IS2_CP	A1
				C. krusei	NEG	0300010001	Oral cavity	F17_T2_OC_CK	B1
52.	F18	27	Twin 1	*C. dubliniensis*	POS	7042100015	Oral cavity	F18_T1_OC1_CD	A2
					POS	7142100015		F18_T1_OC2_CD	A1
				C. guilliermondii	NEG	7577352117	Interdigital space	F18_T1_ IS1_CG	A1
					NEG	5577352117		F18_T1_ IS2_CG	A2
53.			Twin 2	*C. dubliniensis*	POS	7142100015	Oral cavity	F18_T2_OC1_CD	A1
					POS	7142100015		F18_T2_OC2_CD	A1
				C. guilliermondii	NEG	5577352117	Interdigital space	F18_T2_ IS_CG	A1
54.	F19	47	Mother	*C. guilliermondii*	NEG	5577352115	Oral cavity	F19_M_OC_CG	C3
					NEG	7577350517	Anus	F19_M_A_CG	C3
					NEG	5577352115	Interdigital space	F19_M_IS_CG	C3
55.		11	Twin 1	*C. dubliniensis*	POS	6142100015	Oral cavity	F19_T1_OC_CD	B1
					POS	7042100015	Anus	F19_T1_A_CD	B2
				C. guilliermondii	NEG	5577352117	Oral cavity	F19_T1_OC_CG	C3
					NEG	5577352117	Anus	F19_T1_A1_CG	C1
					NEG	7577352117		F19_T1_A2_CG	Un1
					NEG	5577352117	Interdigital space	F19_T1_ IS_CG	C1
					NEG	5577352115	Navel	F19_T1_N_CG	C1
56.			Twin 2	*C. dubliniensis*	POS	6142100015	Oral cavity	F19_T2_OC1_CD	B2
					POS	7042100015		F19_T2_OC2_CD	B2
57.	F20	50	Twin 1	*C. dubliniensis*	POS	7142100015	Anus	F20_T1_A1_CD	C1
					POS	6142100015		F20_T1_A2_CD	Un1

58.			Twin 2	*C. dubliniensis*	POS	7142100015	Oral cavity	F20_T2_OC_CD	C2
59.	F21*	67	Twin 1	*C. albicans*	POS	7146340015	Oral cavity	F21_T1_OC_CA	H
					POS	7146340015	Anus	F21_T1_A_CA	H
				C. guilliermondii	NEG	5577352115	Anus	F21_T1_A_CG	C1
60.			Twin 2	*C. albicans*	POS	7347340015	Oral cavity	F21_T2_OC_CA	H
				C. guilliermondii	NEG	5577352115	Oral cavity	F21_T2_OC_CG	C3
61.	F22*	34	Twin 1	*C. albicans*	POS	7147340015	Oral cavity	F22_T1_OC_CA	F3
62.			Twin 2	*C. albicans*	POS	7147340015	Oral cavity	F22_T2_OC_CA	F3
63.	F23	36	Mother	*C. albicans*	POS	7147340015	Oral cavity	F23_M_OC1_CA	G2
					POS	7147340015		F23_M_OC2_CA	G1
					POS	7147340015		F23_M_OC3_CA	G1
64.		8	Twin 1	*C. guilliermondii*	NEG	5577352117	Interdigital space	F23_T1_ IS_CG	C2
65.			Twin 2	*C. albicans*	POS	7143340015	Oral cavity	F23_T2_OC_CA	G1
				C. guilliermondii	NEG	5577352117	Interdigital space	F23_T2_ IS_CG	C2
66.	F24	26	Twin 1	*C. albicans*	POS	7347350215	Oral cavity	F24_T1_OC1_CA	K4
					POS	7347350215		F24_T1_OC2_CA	K4
					POS	7347350215	Anus	F24_T1_A1_CA	K4
					POS	7347350215		F24_T1_A2_CA	K4
					POS	7347350215		F24_T1_A3_CA	K4
67.	F25*	34	Father	*C. albicans*	POS	7146340015	Oral cavity	F25_F_OC1_CA	K2
					POS	7146340015		F25_F_OC2_CA	K2
68.		2	Twin 1	*C. albicans*	POS	7146340015	Oral cavity	F25_T1_OC_CA	K2
					POS	7146340015	Navel	F25_T1_N_CA	K2
				C. guilliermondii	NEG	5577352117	Oral cavity	F25_T1_OC1_CG	B1
					NEG	5577352117		F25_T1_OC2_CG	B1
					NEG	5577352117		F25_T1_OC3_CG	B1
69.			Twin 2	*C. albicans*	POS	7146340015	Oral cavity	F25_T2_OC_CA	K2
				C. guilliermondii	NEG	5577352117	Oral cavity	F25_T2_OC_CG	B1
70.	F26	24	Twin 1	*C. parapsilosis*	NEG	5547350717	Oral cavity	F26_T1_OC_CP	F1
					NEG	5547350717	Anus	F26_T1_A_CP	F1
					NEG	7547350317	Interdigital space	F26_T1_ IS_CP	F1
71.			Twin 2	*C. albicans*	POS	7347340015	Oral cavity	F26_T2_OC_CA	K2
					POS	7347340015	Anus	F26_T2_A1_CA	K2
					POS	7347340015		F26_T2_A2_CA	K2
72.	F27	27	Mother	*C. albicans*	POS	7346340015	Oral cavity	F27_M_OC_CA	K1
				C. parapsilosis	NEG	5547350311	Interdigital space	F27_M_ IS_CP	I2
73.		1	Twin 1	*C. parapsilosis*	NEG	5547350311	Interdigital space	F27_T1_ IS_CP	I1
74.			Twin 2	*C. albicans*	POS	7346340015	Oral cavity	F27_T2_OC1_CA	K1
					POS	7346340015		F27_T2_OC2_CA	K1
75.	F28	40	Mother	*C. albicans*	POS	7347350215	Oral cavity	F28_M_OC1_CA	K3

					POS	7347350215		F28_M_OC2_CA	K3
					POS	7347350215		F28_M_OC3_CA	K2
					POS	7347350215	Anus	F28_M_A_CA	K3
76.		17	Twin 1	*C. albicans*	POS	7347350215	Oral cavity	F28_T1_OC_CA	K3
77.			Twin 2	*C. albicans*	POS	7347350215	Oral cavity	F28_T2_OC_CA	K2
					POS	7347350215		F29_T1_OC1_CA	E2
					POS	7347350215		F29_T1_OC2_CA	E2
78.			Twin 1	*C. albicans*	POS	7347350215	Oral cavity	F29_T1_OC3_CA	E2
	F29	15			POS	7347350215		F29_T1_OC4_CA	E2
					POS	7347350215		F29_T1_OC5_CA	E1
					POS	7347340015	Anus	F29_T1_A_CA	E2
79.			Twin 2	*C. albicans*	POS	7347350215	Oral cavity	F29_T2_OC_CA	E2
80.			Twin 1	*C. parapsilosis*	NEG	5547350317	Oral cavity	F30_T1_OC_CP	G1
	F30	13			NEG	5547350313	Interdigital space	F30_T1_ IS_CP	F2
81.			Twin 2	*C. parapsilosis*	NEG	5547350313	Oral cavity	F30_T2_OC1_CP	G1
					NEG	5547350313		F30_T2_OC2_CP	G2
82.		27	Mother	*C. parapsilosis*	NEG	5547350313	Anus	F31_M_A_CP	J
83.		29	Father	*C. parapsilosis*	NEG	5547350317	Anus	F31_F_A_CP	J
84.	F31		Twin 1	*C. parapsilosis*	NEG	5547350313	Oral cavity	F31_T1_OC_CP	J
85.		2	Twin 2	*C. parapsilosis*	NEG	5547350317	Oral cavity	F31_T2_OC_CP	J
					NEG	5547350313	Interdigital space	F31_T2_ IS_CP	Un4
88.			Twin 1	*C. albicans*	POS	7143340015	Oral cavity	F32_T1_OC1_CA	L3
	F32*	44			POS	7346340015		F32_T1_OC2_CA	L2
87.			Twin 2	*C. albicans*	POS	7346340015	Oral cavity	F32_T2_OC_CA	L1
88.			Twin 1	*C. guilliermondii*	NEG	7577350517	Interdigital space	F33_T1_ IS_CG	C2
	F33	9			NEG	7577350517		F33_T2_ IS1_CG	C2
89.			Twin 2	*C. guilliermondii*	NEG	7547350317	Interdigital space	F33_T2_ IS2_CG	C2

The genotypes of the different *Candida* strains are marked with the following colors: yellow – *Candida albicans*, blue – *Candida parapsilosis*, green – *Candida guilliermondii*, red – *Candida dubliniensis*, gray – *Candida krusei*. POS – positive, NEG – negative. * – family in which affirmed occurrence monozygotic twins.

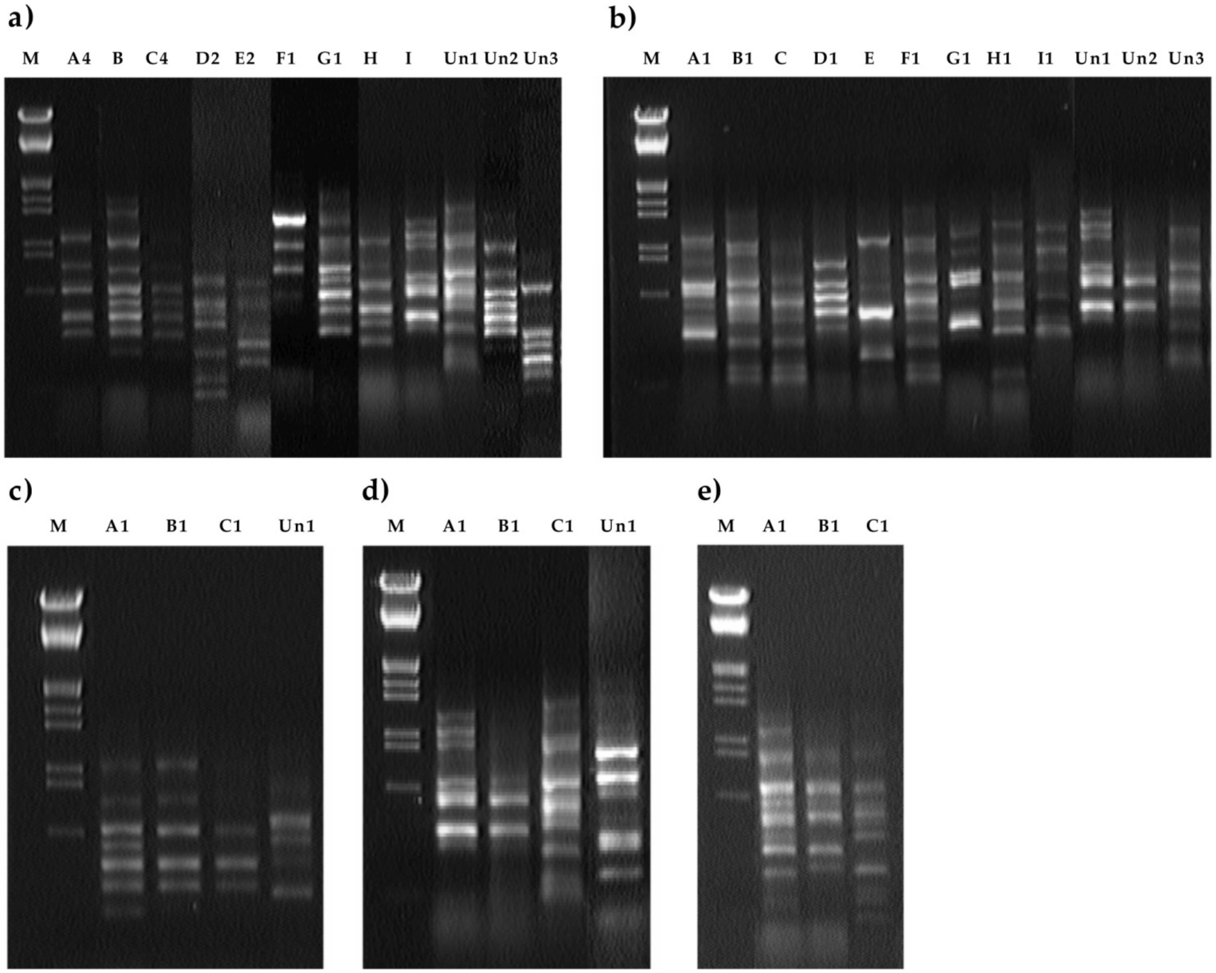

Figure S1. The representative examples of dendrograms for: a) *Candida albicans*, b) *C. parapsilosis*, c) *C. guilliermondii*, d) *C. dubliniensis* and e) *C. krusei*. M – marker.
